# Supplementary material for: Assessing dynamic functional connectivity in heterogeneous samples
Source: Neuroimage. 2017 Aug 15;157:635–47. doi: 10.1016/j.neuroimage.2017.05.065 (PMC5590721; doi:10.1016/j.neuroimage.2017.05.065)
Supplement: Application 1 [file mmc1.pdf]

Assessing dynamic functional connectivity in heterogeneous samples
Supplementary Material

Effect of window length on k-means simulation

In the main text, we report results of the k-means analysis for window length $w = 30$. The effect of window length has previously been investigated by Shakil et al. (2016). The results of the k-means simulation for window lengths $w = 60, 90, 120$ can be seen in Figures S1, S2 and S3). These produced very similar results to the $w = 30$ analysis and support our conclusion that misspecification of the number of FC states k in a dFC k-means analysis can result in spurious group differences in the number of detected FC state transitions. Figure S4 directly compares the mean centroid errors for different window lengths. While longer window lengths result in lower centroid errors for small k, shorter windows result in a better estimation of the FC states when k is close to the true number of FC states ($k = 9$ for G1, $k = 6$ for G2). In particular, the most accurate estimate of FC states occurs for shortest window length, $w = 30$, when k is correctly specified.

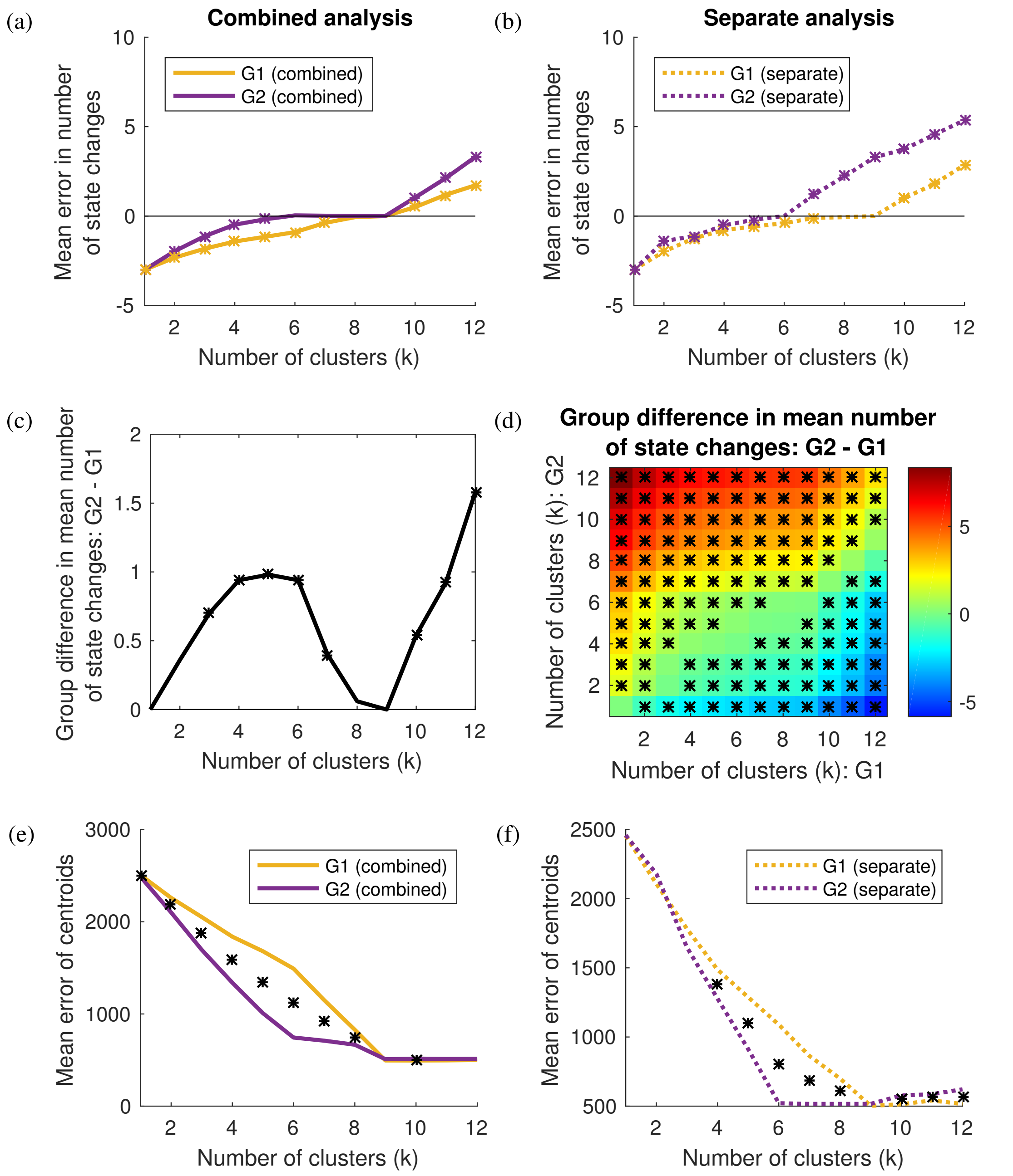

Figure S1: The results for the k-means simulation for window length $w = 60$. The true number of FC state transitions in this simulation is 3. Individuals in G1 could reach 9 FC states while individuals in G2 could reach only 6 of these 9 FC states. (a) When $k < 9$, the combined analyses underestimated the number of FC state transitions for G1 (solid yellow line), while the number of FC state transitions for G2 (solid purple line) was recovered more accurately. (b) The separate analysis showed an improvement in the error in number of FC state transitions for G1 (dotted yellow line) when k was underestimated. (c) Unless k was correctly estimated, the combined analysis yielded an incorrect group difference in number of FC state transitions. (d) For the separate analysis, incorrect group differences were only found when k was overestimated or grossly misspecified for either group. (e) In terms of recovered space-time connectivity structure, the combined analysis performed better for G2 (solid purple line) than G1 (solid yellow line) when k was underestimated. (f) If k was correctly specified or only slightly misspecified for either group, the separate analyses had a similar error in recovered connectivity structure. Asterisks in (a-d) indicate a statistically significant ($p < 0.05$) difference from zero, according to a Wilcoxon signed-rank test, while asterisks in (e-f) indicate a statistically significant ($p < 0.05$) difference between the two groups, according to a two-sample Wilcoxon rank-sum test.

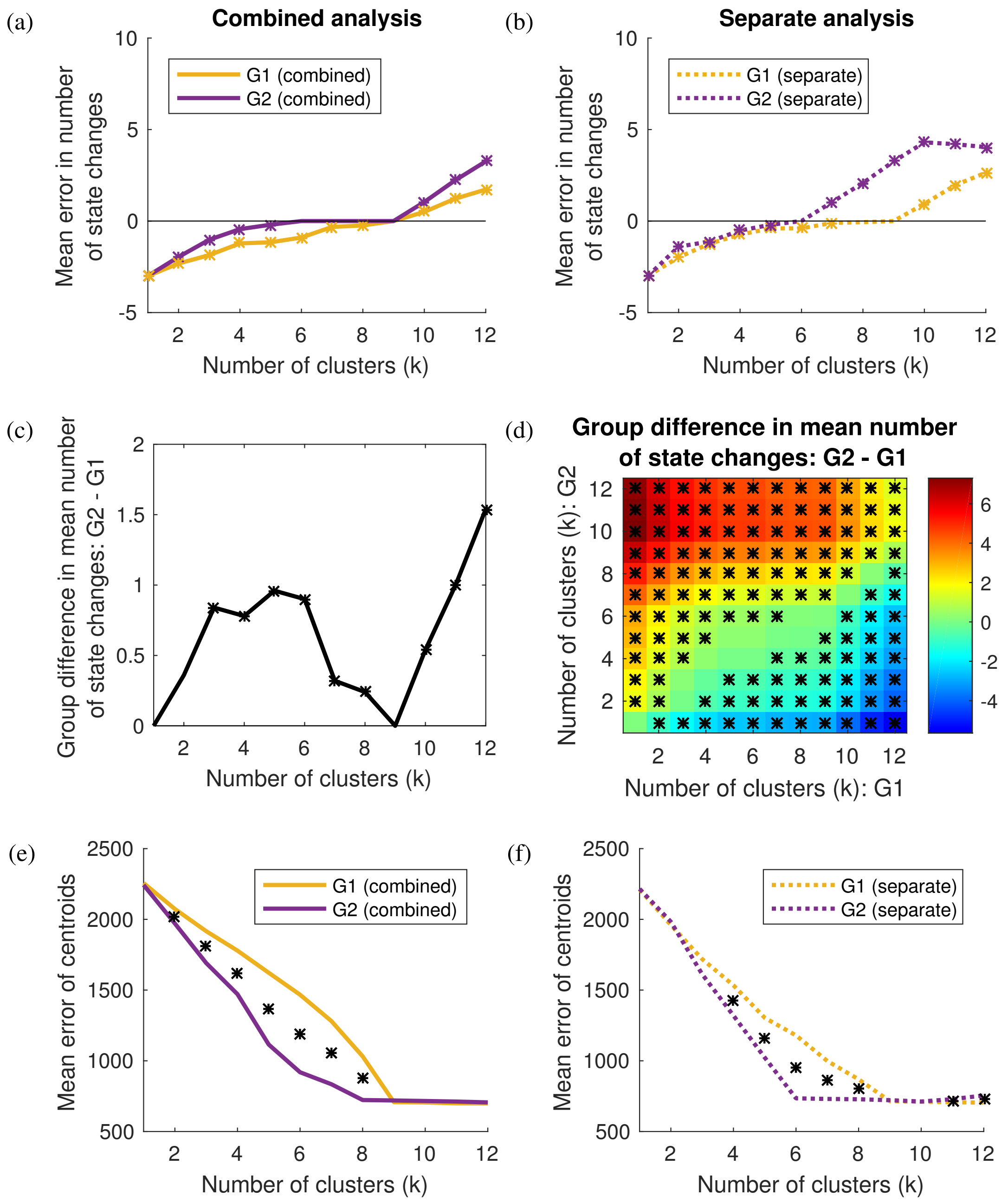

Figure S2: The results for the k-means simulation for window length $w = 90$. The true number of FC state transitions in this simulation is 3. Individuals in G1 could reach 9 FC states while individuals in G2 could reach only 6 of these 9 FC states. (a) When $k < 9$, the combined analyses underestimated the number of FC state transitions for G1 (solid yellow line), while the number of FC state transitions for G2 (solid purple line) was recovered more accurately. (b) The separate analysis showed an improvement in the error in number of FC state transitions for G1 (dotted yellow line) when k was underestimated. (c) Unless k was correctly estimated, the combined analysis yielded an incorrect group difference in number of FC state transitions. (d) For the separate analysis, incorrect group differences were only found when k was overestimated or grossly misspecified for either group. (e) In terms of recovered space-time connectivity structure, the combined analysis performed better for G2 (solid purple line) than G1 (solid yellow line) when k was underestimated. (f) If k was correctly specified or only slightly misspecified for either group, the separate analyses had a similar error in recovered connectivity structure. Asterisks in (a-d) indicate a statistically significant ($p < 0.05$) difference from zero, according to a Wilcoxon signed-rank test, while asterisks in (e-f) indicate a statistically significant ($p < 0.05$) difference between the two groups, according to a two-sample Wilcoxon rank-sum test.

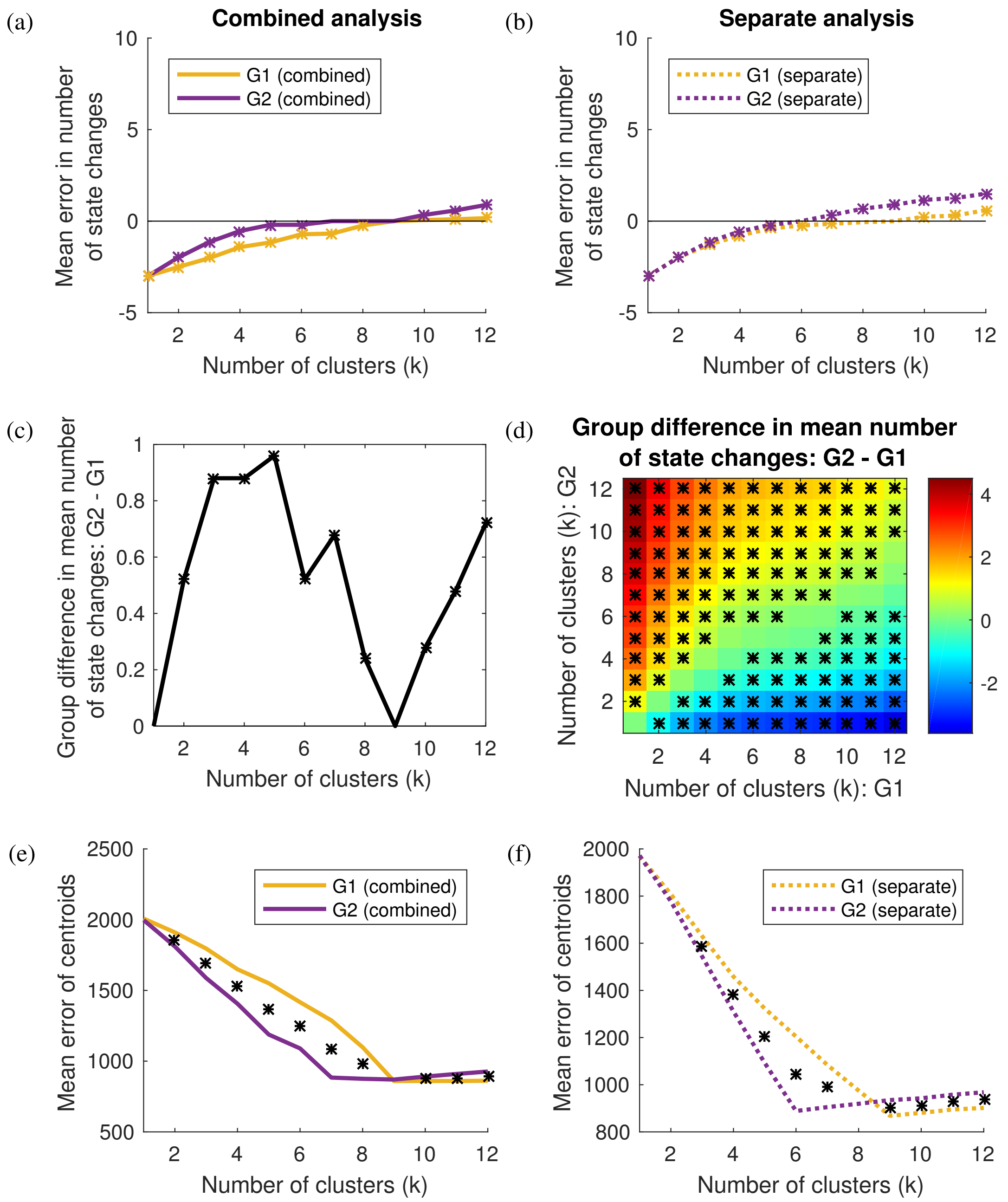

Figure S3: The results for the k-means simulation for window length $w = 120$. The true number of FC state transitions in this simulation is 3. Individuals in G1 could reach 9 FC states while individuals in G2 could reach only 6 of these 9 FC states. (a) When $k < 9$, the combined analyses underestimated the number of FC state transitions for G1 (solid yellow line), while the number of FC state transitions for G2 (solid purple line) was recovered more accurately. (b) The separate analysis showed an improvement in the error in number of FC state transitions for G1 (dotted yellow line) when k was underestimated. (c) Unless k was correctly estimated, the combined analysis yielded an incorrect group difference in number of FC state transitions. (d) For the separate analysis, incorrect group differences were only found when k was overestimated or grossly misspecified for either group. (e) In terms of recovered space-time connectivity structure, the combined analysis performed better for G2 (solid purple line) than G1 (solid yellow line) when k was underestimated. (f) If k was correctly specified or only slightly misspecified for either group, the separate analyses had a similar error in recovered connectivity structure. Asterisks in (a-d) indicate a statistically significant ($p < 0.05$) difference from zero, according to a Wilcoxon signed-rank test, while asterisks in (e-f) indicate a statistically significant ($p < 0.05$) difference between the two groups, according to a two-sample Wilcoxon rank-sum test.

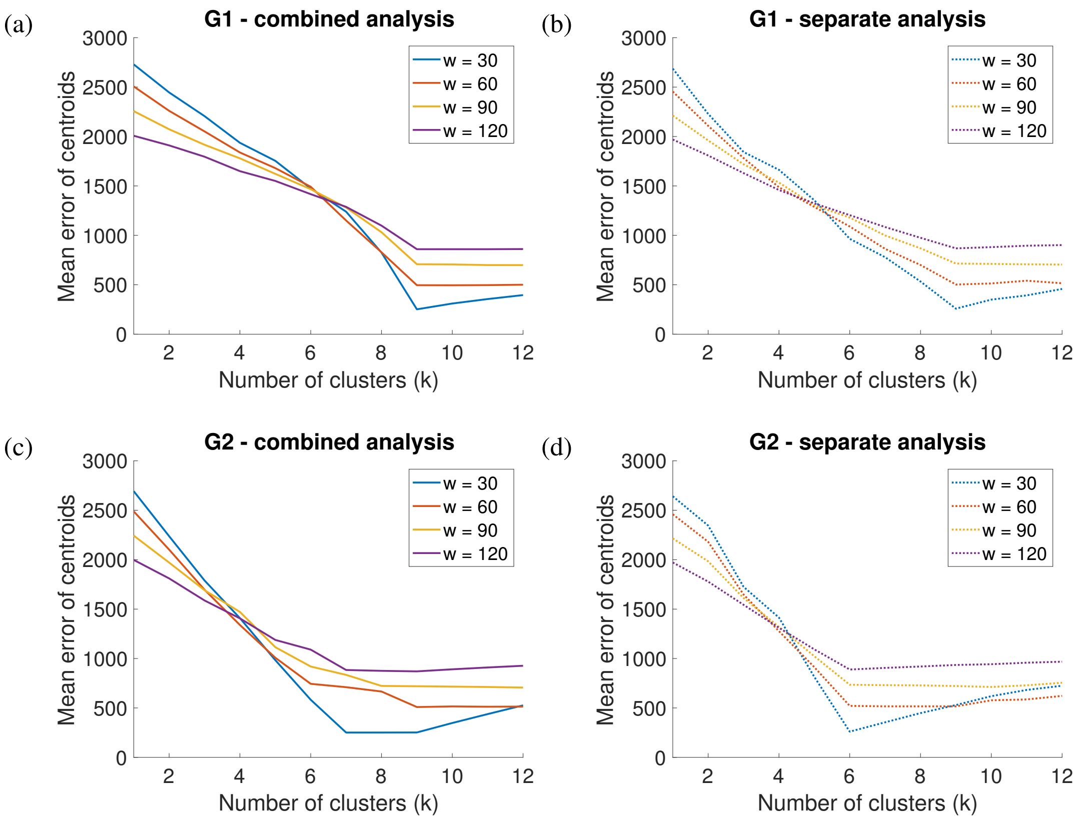

Figure S4: The effect of window length on mean (across subjects) error of centroids in the k-means simulation for (a) G1 in the combined analysis, (b) G1 in the separate analysis, (c) G2 in the combined analysis, and (d) G2 in the separate analysis. ndividuals in G1 could reach 9 FC states while individuals in G2 could reach only 6 of these 9 FC states. Window length $w = 30$ is reported in the main text. These results show that when k is correctly estimated or only slightly misspecified, a longer window results in a worse estimation of the FC states, as evidenced by the increased mean centroid error.

Parameter variation

In the main text, we reported results for a single choice of various parameters. Namely, we fixed the equilibrium probability of module-specific events at $P_{mod} = 0.5$, the equilibrium probability of region-specific events at $P_{reg} = 0.5$, the autocorrelation of region-specific events at $\rho_{reg} = 0$, the amplitude of module-specific events relative to region-specific events at $a_{mod} = 2$, and the standard deviation of white noise at σ_{noise}. Note that we vary the latter three parameters in Sections 3.1, 3.3, and 3.4 respectively.

Here, we report a summary of the results for a wide range of these parameter values. Figure S5 shows the results of the neural autocorrelation simulation described in Section 3.1 for $P_{mod} = 0.25, 0.5, 0.75$, $P_{reg} = 0.25, 0.5, 0.75$, $a_{mod} = 1, 2, 3$, and $\sigma_{noise} = 0.2, 0.5, 1$. Figure S7 shows the results of the measurement noise simulation described in Section 3.4 for $P_{mod} = 0.25, 0.5, 0.75$, $P_{reg} = 0.25, 0.5, 0.75$, $\rho_{reg} = -0.5, 0, 0.5$, and $a_{mod} = 1, 2, 3$. Figure S6 shows the results of the connectivity strength described in Section 3.3 for $P_{mod} = 0.25, 0.5, 0.75$, $P_{reg} = 0.25, 0.5, 0.75$, $\rho_{reg} = -0.5, 0, 0.5$, and $\sigma_{noise} = 0.2, 0.5, 1$. For each combination of parameter values, we determine whether or not the parameter of interest x had an impact on estimated dFC σ_{corr} by fitting the linear model $\sigma_{corr} = \alpha + \beta_1 x + \beta_2 x^2 + \epsilon$ where $\epsilon \sim \mathcal{N}(0, \sigma^2)$. The statistical significance of the effect of x or x^2 on σ_{corr} is determined by whether the 95% confidence interval for the corresponding coefficient contains zero. Thus the parameter of interest has a statistically significant impact on estimated dFC if at least one of the confidence intervals for x or x^2 does not contain zero. We indicate statistically significant effect by a black square.

Figure S5 shows that neural autocorrelation impacted estimated dFC between dynamically connected regions for the whole explored parameter space while the impact of neural autocorrelation on estimated dFC between the statically connected regions for all the parameter values differed throughout the parameter space. Similarly, Figure S6 shows that connectivity strength had a statistically significant impact on estimated dFC between dynamically connected regions for all the parameter values investigated, but the effect on statically connected regions depends on the choice of parameters. Figure S7 shows that measurement noise had a significant effect on estimated dFC between all three connectivity types for the whole explored parameter space. These results demonstrate that neural autocorrelation, connectivity strength and measurement noise have an impact on estimated dFC for a wide range of parameters, despite the underlying dynamic connectivity structure remaining constant.

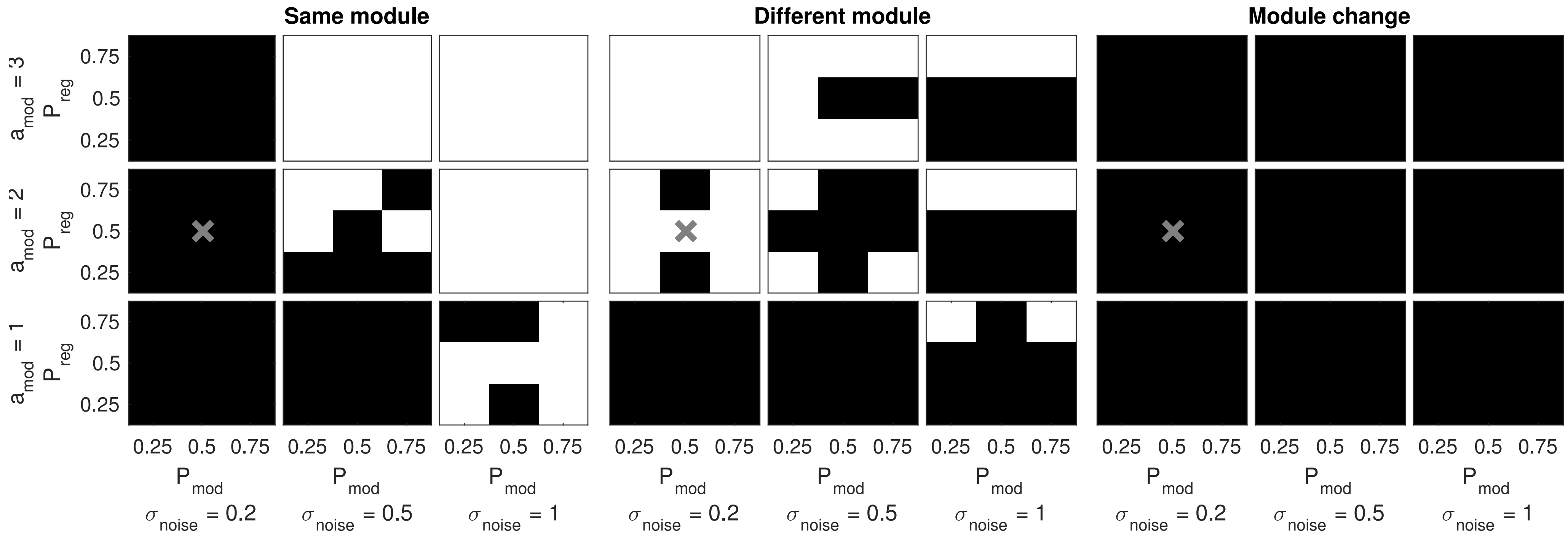

Figure S5: The effect of neural autocorrelation on estimated dFC for a range of parameter values. To assess the impact of neural autocorrelation ρ_{reg} on estimated dFC ,σ_{corr}, we fit the linear model: $\sigma_{corr} = \alpha + \beta_1\rho_{reg} + \beta_2\rho_{reg}^2 + \epsilon$ where $\epsilon \sim \mathcal{N}(0, \sigma^2)$. The statistical signifance of the effect of ρ_{reg} or ρ_{reg}^2 on σ_{corr} is determined by whether the 95% confidence interval for the corresponding coefficient contains zero. Thus neural autocorrelation has a statistically signifcant impact on estimated dFC if at least one of the confidence intervals for ρ_{reg} or ρ_{reg}^2 does not contain zero. A statistically signifcant effect is indicated by a black square. We considered three values each of the equilibrium probability of module-specific events P_{mod}, the equilibrium probability of region-specific events P_{reg}, the autocorrelation of region-specific events ρ_{reg}, and the standard deviation of white noise σ_{noise}. A grey cross indicates the parameter values used in the main text.

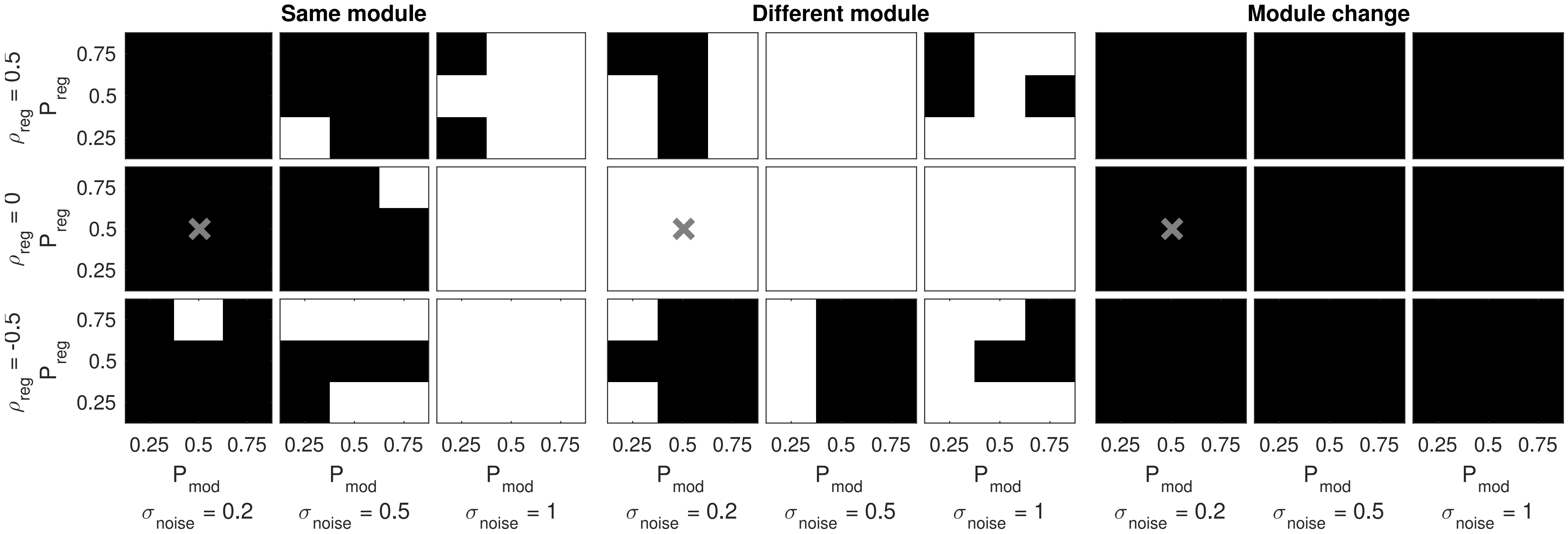

Figure S6: The effect of connectivity strength on estimated dFC for a range of parameter values. To assess the impact of connectivity strength a_{mod} on estimated dFC ,σ_{corr}, we fit the linear model: $\sigma_{corr} = \alpha + \beta_1 a_{mod} + \beta_2 a_{mod}^2 + \epsilon$ where $\epsilon \sim \mathcal{N}(0, \sigma^2)$. The statistical signifance of the effect of a_{mod} or a_{mod}^2 on σ_{corr} is determined by whether the 95% confidence interval for the corresponding coefficient contains zero. Thus connectivity strength has a statistically signifcant impact on estimated dFC if at least one of the confidence intervals for a_{mod} or a_{mod}^2 does not contain zero. A statistically signifcant effect is indicated by a black square. We considered three values each of the equilibrium probability of module-specific events P_{mod}, the equilibrium probability of region-specific events P_{reg}, the autocorrelation of region-specific events ρ_{reg}, and the standard deviation of white noise σ_{noise}. The grey cross indicates the parameter values used in the main text.

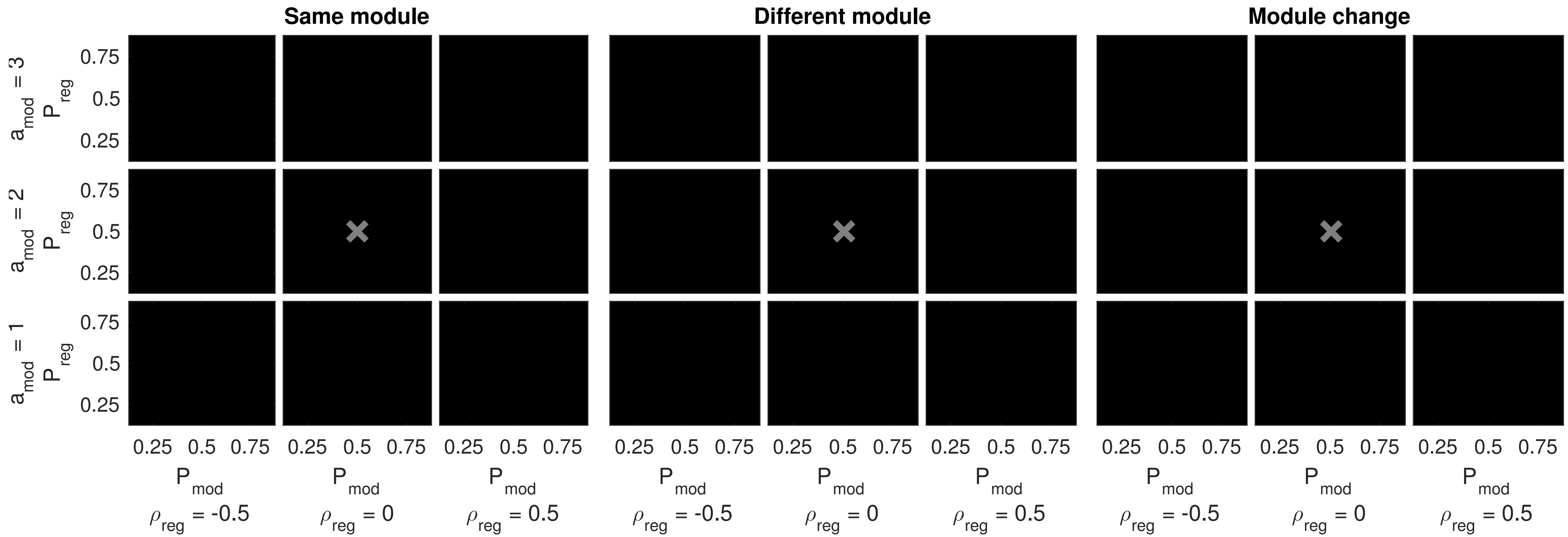

Figure S7: The effect of measurement noise on estimated dFC for a range of parameter values. To assess the impact of measurement noise σ_{noise} on estimated dFC ,σ_{corr}, we fit the linear model: $\sigma_{corr} = \alpha + \beta_1 \sigma_{noise} + \beta_2 \sigma^2_{noise} + \epsilon$ where $\epsilon \sim \mathcal{N}(0, \sigma^2)$. The statistical signifance of the effect of σ_{noise} or σ^2_{noise} on σ_{corr} is determined by whether the 95% confidence interval for the corresponding coefficient contains zero. Thus measurement noise has a statistically signifcant impact on estimated dFC if at least one of the confidence intervals for σ_{noise} or σ^2_{noise} does not contain zero. A statistically signifcant effect is indicated by a black square. We considered three values each of the equilibrium probability of module-specific events P_{mod}, the equilibrium probability of region-specific events P_{reg}, the autocorrelation of region-specific events ρ_{reg}, and the amplitude of module-specific events a_{mod}. A grey cross indicates the parameter values used in the main text.

Impact of prewhitening on neural autocorrelation results

In the simulations, we do not prewhiten the fMRI time series prior to our dynamic connectivity analysis. While the impact of prewhitening on static functional connectivity has already been studied by Arbabshirani et al. (2014), the effect of prewhitening on dynamic connectivity is not as well evidenced. Here, we include the results of the neural autocorrelation simulation (see Section 3.1) after prewhitening. The details of the prewhitening procedure can be found in Geerligs et al. (2016). Figure S8 shows that neural autocorrelation impacts estimated dFC even after prewhitening. S8c shows that although pre-whitening attenuates the negative effect of increasing neural autocorrelation on dFC estimates for truly dynamic connections, a significant negative effect of neural autocorrelation remains after pre-whitening. Furthermore, S8a shows that the positive effect of increasing neural autocorrelation on dFC estimates for within-module static connections actually becomes a significant negative effect after pre-whitening. Figure S8b shows that for the unconnected regions, estimated dFC increases as neural autocorrelation increases from -0.8 to 0, but decreases from 0 to 0.8. Thus pre-whitening does not fully solve the problem.

We do not perform pre-whitening on the remaining analyses in the main paper for the following reasons. Firstly, pre-whitening is actually rarely done for resting-state connectivity analyses (see Arbabshirani et al. (2014)). Secondly, our pre-whitening, which is based on proper restricted maximum likelihood estimation of the simultaneous cross-correlation and residual auto-correlation, requires efficient estimation of the temporal covariance of the data. In real data, the efficiency of estimating the temporal covariance matrix can be improved by pooling across voxels (within an ROI for example). Because we only simulate single time-series for each region in our model, our pre-whitening will be inefficient (noisy). Thirdly, our pre-whitening assumes stationarity of the time-series autocorrelation, and to estimate dynamic (window-specific) autocorrelation would be even less efficient. A full investigation of the effect of prewhitening on dynamic functional connectivity is beyond the scope of this work, although this topic deserves a more in-depth study.

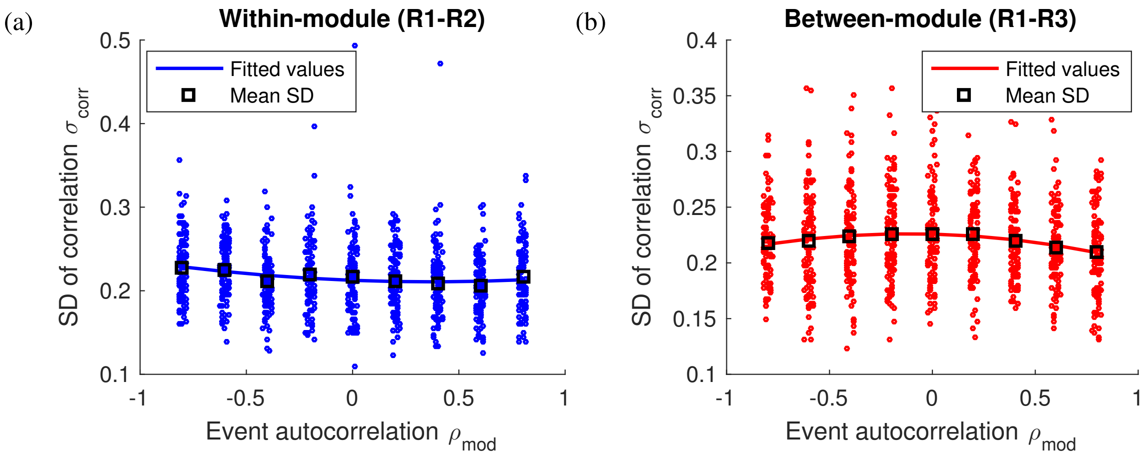

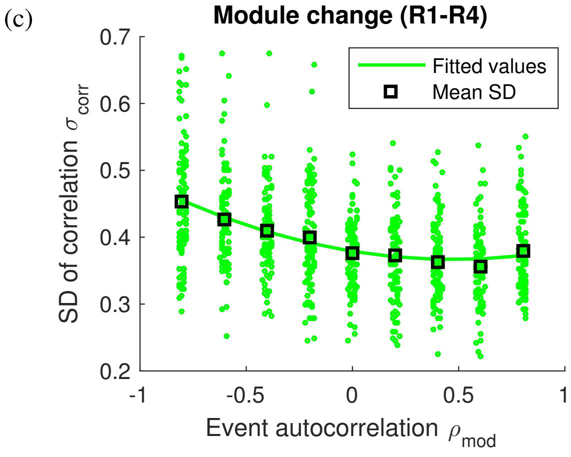

(d)

Regression results for dFC

Connection type	Covariate	Estimate	95% CI
Within-module	ρ_{mod}	-0.010	**(-0.015, -0.005)**
	ρ^2_{mod}	0.013	**(0.002, 0.024)**
Between-module	ρ_{mod}	-0.005	(-0.010, 0.000)
	ρ^2_{mod}	-0.020	**(-0.031, -0.009)**
Module change	ρ_{mod}	-0.051	**(-0.060, -0.043)**
	ρ^2_{mod}	0.054	**(0.035, 0.073)**

Model: $\sigma_{corr} = \alpha + \beta_1\rho_{mod} + \beta_2\rho^2_{mod} + \epsilon$
$\epsilon \sim \mathcal{N}(0, \sigma^2)$

Figure S8: The impact of neural autocorrelation on estimated dFC after prewhitening, measured by the SD of the correlation time series, between (a) statically connected regions (within-module), (b) unconnected regions (between-module), and (c) dynamically connected regions (module change), with (d) the results of a multiple regression. Ri refers to Region i. Region pair R1-R4 has a dynamic connection so the true dFC should be higher than region pairs R1-R2 and R1-R3, which have a static connection. Estimated dFC between statically connected (within-module) regions decreases slightly with neural autocorrelation, while estimated dFC for dynamically connected regions (module change) decreased with increased neural autocorrelation. The impact of neural autocorrelation on estimated dFC is dampened when prewhitening is performed compared to when the simulated time series are not prewhitened (compare to Figure 3 in main text). The neural autocorrelation is varied independently of the underlying connectivity structure, so changing it should have no effect on dFC. The multiple regression assesses the impact of neural autocorrelation on estimated dFC with a statistically significant effect indicated by a 95% confidence interval (CI) in bold type. The solid lines in (a-c) correspond to the fitted values of the multiple regression.

References

Arbabshirani, M. R., Damaraju, E., Phlypo, R., Plis, S., Allen, E., Ma, S., Mathalon, D., Preda, A., Vaidya, J. G., Adali, T., et al., 2014. Impact of autocorrelation on functional connectivity. Neuroimage 102, 294–308.

Geerligs, L., Cam-CAN, Henson, R. N., 2016. Functional connectivity and structural covariance between regions of interest can be measured more accurately using multivariate distance correlation. NeuroImage 135, 16 – 31.
URL http://www.sciencedirect.com/science/article/pii/S1053811916300878

Shakil, S., Lee, C.-H., Keilholz, S. D., 2016. Evaluation of sliding window correlation performance for characterizing dynamic functional connectivity and brain states. NeuroImage 133, 111 – 128.
URL http://www.sciencedirect.com/science/article/pii/S1053811916001920
